# Optimization of the Selective Monohydrolysis of Diethyl 4-Aryl-4*H*-pyran-3,5-dicarboxylates

**DOI:** 10.3390/molecules16053845

**Published:** 2011-05-06

**Authors:** Jiaojiao Duan, Xiaohui Song, Hong Yan, Xiuqing Song

**Affiliations:** College of Life Science and Bio-engineering, Beijing University of Technology, Pingleyuan Street Number 100, Chaoyang District, Beijing 100124, China

**Keywords:** monohydrolysis, 4-aryl-4*H*-pyran, quaternary ammonium salt, monoester, symmetric diester

## Abstract

A simple, efficient and eco-friendly procedure for the selective monohydrolysis of diethyl 2,6-dimethyl-4-aryl-4*H*-pyran-3,5-dicarboxylates under quaternary ammonium salt catalysis conditions is presented. The catalytic activities of various quaternary ammonium salts were investigated using different molar ratios of NaOH and water-organic solvent mixtures. The results indicate that the combination of 1.0 equivalent of tetraethyl-ammonium bromide (TEAB) with 1.2 equivalents of NaOH in a 10% water-ethanol media at 40 °C displays remarkable selectivity for the monohydrolysis of diethyl 2,6-dimethyl-4-aryl-4*H*-pyran-3,5-dicarboxylates. The utility of this process is demonstrated by the monohydrolysis of a series of 4-aryl-4*H*-pyran-3,5-dicarboxylate esters to afford the corresponding monoesters in 20–80% yields under the optimized conditions.

## 1. Introduction

The 4*H*-pyran moiety represents a privileged structure because many of its derivatives possess useful pharmacological activities [1,2]. In recent years, polyfunctionalized 4*H*-pyrans and their derivatives have been the subject of significant interest from the synthetic community and have been widely recognized as versatile scaffolds with diverse biological activities [3,4]. Diethyl 2,6-dimethyl-4-aryl-4*H*-pyran-3,5-dicarboxylates **1** represent an important class of 4*H*-pyrans that is widely used in therapeutic areas, especially for the inhibition of the HIV-1 proteases [5,6,7,8]. The mono-carboxylic acids, or monoesters **2** of compounds **1** hold potential biological activities similar to the biological activity of **1**, and are very versatile building blocks in organic synthesis. Diethyl 2,6-dimethyl-4-aryl-4*H*-pyran-3,5-dicarboxylates **1** have routinely been efficiently synthesized by the reaction of aryl aldehydes and 1,3-diketones catalyzed by ZnCl_2_ under ultrasound irradiation [9]. Here, we report the synthesis of 4H-pyran hemiesters **2** by the selective monohydrolysis of symmetric diesters **1**.

**Scheme 1:**
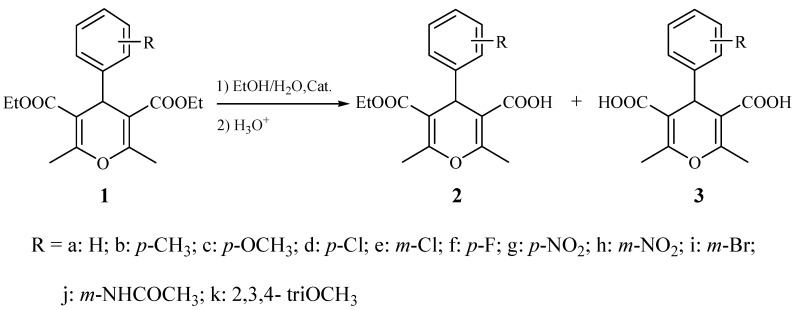
Formation of **2** from **1** in the presence of TEAB catalyst.

Ester hydrolysis is one of the most fundamental reactions in organic chemistry and commonly referred to as alkaline hydrolysis or saponification. This method, using a base such as NaOH in aqueous alcohol, has proven highly reliable for the hydrolysis of a single ester into a carboxylic acid on large scales and at low cost. Unfortunately, it usually does not afford good results when applied to diesters. In that case it typically produces complex mixtures of diacids, monoesters and the starting diesters, and the separation of these product mixtures is often very difficult. One effective method for the monohydrolysis of symmetric diesters utilizes enzymes [10,11,12,13]. The monohydrolysis of symmetric diesters without the use of enzymes has proven to be a more challenging task. The only previously reported non-enzymatic, selective monohydrolysis utilizes an aqueous NaOH solution with THF and water as co-solvents [14,15,16,17]. Recently, quaternary ammonium salts have emerged as useful catalysts in a number of organic transformations. Their cost, ready availability and inherent properties, such as environmental compatibility, greater selectivity, operational simplicity, non-corrosive nature and ease of reusability, make them highly practical choices for many applications [18,19,20]. We therefore became interested in examining the behavior of quaternary ammonium ions as catalysts for the selective monohydrolysis of diethyl 2,6-dimethyl-4-aryl-4*H*-pyran-3,5-dicarboxylates **1** in water-mediated solvents (Scheme 1).

## 2. Results and Discussion

In order to study the behavior of quaternary ammonium salts as catalysts for the selective monohydrolysis of diethyl 2,6-dimethyl-4-aryl-4*H*-pyran-3,5-dicarboxylates **1**, and to determine the optimal conditions with regard to the types of catalyst, equivalents of NaOH, solvents and reaction temperature, an initial model study was carried out on the monohydrolysis of diethyl 2,6-dimethyl-4-aryl-4*H*-pyran-3,5-dicarboxylate (**1a**).

The solvents ethanol and acetone used in classical ester hydrolysis were chosen for monohydrolysis of **1a**, and tetrahydrofuran and acetonitrile were also used in the co-solvent hydrolysis. The conditions below were ascertained according to the reference [21,22,23]. These results presented in Table 1 were obtianed under the conditions of 1.2 equivalents of NaOH and 1.0 equivalent of tetraethyl-ammonium bromide (TEAB) as a catalyst in a 10% aqueous-organic solvent system at 40 °C.

**Table 1:** Solvent effects on the monohydrolysis of **1a** using 1.2 equivalents of NaOH.

Entry	Solvents	Temperature (°C)	Time (h)	Ratio ^a^ 2a:3a	Yield ^b^ (%) 2a
1	Tetrahydrofuran	25	4.5	61:39	46
2	Tetrahydrofuran	40	5.0	70:30	50
3	Tetrahydrofuran	55	5.0	63:37	47
4	Ethanol	25	4.0	78:22	62
5	Ethanol	40	4.0	81:19	80
6	Ethanol	55	4.0	75:25	63
7	Acetone	25	5.0	42:58	35
8	Acetone	40	5.0	50:50	40
9	Acetone	55	5.0	45:55	38
10	Acetonitrile	25	6.0	36:64	25
11	Acetonitrile	40	5.5	41:59	32
12	Acetonitrile	55	6.0	34:66	23

^a^ Ratios based on HPLC analysis; ^b^ Isolated yields of **2a** recrystallization from acetone.

In the monohydrolysis of **1a**, the product ratios of **2a** and **3a** appeared to be higher in ethanol than the other solvents at 40 °C, showing high partial selectivity. It seemed that the polar protic nature of ethanol was beneficial for higher selectivity, whereas aprotic solvents were not as effective. The efficacy of the ethanol was apparent in these reactions, as it appeared to increase the solubilities of the diester **1** and monoester **2** and more efficiently disperse them in the reaction mixture. Other advantages of ethanol as solvent include that it is somewhat more environmentally friendly and allows for straightforward recovery of the catalyst by filtration through a short column of magnesium sulfate. There was a great reason that ethanol provided a polar environment in which TEAB played a much more obvious effect on monohydrolysis of **1a** than other solvent.

**Table 2:** The effect of water-ethanol percentages on the monohydrolysis of **1a** using 1.2 equivalents of NaOH.

Entry	% Water (w/w%)	Time (h)	Ratio ^a^ 2a:3a	Yield ^b^ (%) 2a
1	0	12.0	46:54	35
2	1	9.0	48:52	38
3	5	5.5	53:47	48
4	8	5.5	74:26	65
5	10	4.0	81:19	80
6	15	3.5	53:47	50
7	20	3.5	36:64	28

^a^ Ratios based on HPLC analysis; ^b^ Isolated yields of **2a** recrystallization from acetone.

Regarding the selective monohydrolysis of **1a**, the effect of the percentage of water (from 0%–20%) in ethanol was also studied with 1.2 equivalents of NaOH and 1.0 equivalent of TEAB at 40 °C, as depicted in Table 2. While increased water content was found to consistently enhance the rate of hydrolysis, the optimal product ratio of **2a** to **3a** was observed in a 10% water-ethanol media. The percentages were below or over 10%, the decreasing ratios of **2a** and **3a** were due to either less or more exposure of the carboethoxy group to the aqueous NaOH, resulting in less reactivity to the diester **1a** and higher reactivity to the diacid **3a**, respectively.

The amount of NaOH used in the monohydrolysis proved to affect the selectivity for monoester **2a** under standard conditions significantly (Table 3). Low amounts of NaOH resulted in lower yields of **2a**, along with lower ratios of **2a** and **3a**. Alternatively, excess NaOH led to increase in diacid **3a**. Interestingly, 1.0 equivalent of NaOH was not enough to maximize the ratio of **2a** and **3a**. In a relatively short reaction time, 1.2 equivalents of NaOH was preferable to 1.5 and 2.0 equivalents of NaOH, whereas the ratio of **2a** and **3a** was higher than those of the 1.5 and 2.0 equivalents of NaOH.

**Table 3:** The effect of NaOH stoichiometry on the monohydrolysis of **1a**.

Entry	NaOH equiv.	Time (h)	Ratio ^a^ 2a:3a	Yield ^b^ (%) 2a
1	0.1	10.0	51:49	34
2	0.3	9.5	52:48	35
3	0.5	8.0	60:40	43
4	1.0	6.0	73:27	70
5	1.2	4.0	81:19	80
6	1.5	4.5	43:57	41
7	2.0	3.5	25:75	23

^a^ Ratios based on HPLC analysis; ^b^ Isolated yields of **2a** recrystallization from acetone.

Four catalysts were used to investigate the effect on the monohydrolysis of **1a**: PEG-400 (polyethylene glycol-400), *β*-CD (*β*-cyclodextrins), tetraethyl-ammonium bromide (TEAB) and tetrabutyl-ammonium bromide (TBAB) under standard conditions (Table 4). 

**Table 4:** The effect of catalysts on the monohydrolysis of **1a** using 1.2 equivalents of NaOH.

Entry	Catalyst	Time (h)	Ratio ^a^ 2a:3a	Yield ^b^ (%) 2a
1	None	12.0	40:60	34
2	PEG-400	8.0	46:54	45
3	β-CD	8.5	37:63	34
4	TEAB	4.0	81:19	80
5	TBAB	4.0	75:25	65

^a^ Ratios based on HPLC analysis; ^b^ Isolated yields of **2a** recrystallization from acetone.

Either in the absence of a catalyst or with PEG-400, *β*-CD and TBAB used as catalyst, the yields of and selectivities for **2a** were lower, with lower **2a**:**3a** ratios. However the purity of **2a** was higher when the reaction was carried out in the presence of TEAB. Only TBAB displayed even comparable catalytic activity to TEAB. Presumably, the shorter alkyl substitutes of TEAB make it more hydrophilic, and thus more soluble, than TBAB, explaining the improved outcome [24]. In this selective monohydrolysis, the mechanisms of TEAB appear to be a surfactant and a cation source of an ion pair. As a surfactant, TEAB forms micelles in 10% water-ethanol media and gives carboxylate anion by coordinating with hydroxide. The subsequent nucleophilic attack forms the carboxyl group functionality and regenerates the TEAB in the aqueous NaOH media. As the cation of an ion pair, TEAB provides Q^+^ in forming ion pairs with the carboxyl anion of monoesters to slow down the second hydrolysis reaction [22].

The amount of TEAB catalyst played a crucial role in the selective monohydrolysis of **1a** under standard conditions (Table 5). The optimal quantity of TEAB proved to be 1.0 equivalent of **1a**, and the purity of **2a** was better in a 80% conversion. The reason was that quaternary cation associated with the carboxylate anion of monoester was more than unassociated with anion. The generation of ion pairs of monoester **2a** with Q^+^ resulted the further hydrolysis of **1a** to diacid **3a** subsequently. When more than 1.0 equivalent of TEAB was used, the ratios of **2a** and **3a** decreased, particularly when greater than 2.0 equivalents of TEAB were used; in this case, the major product was diacid **3a**.

**Table 5:** The effect of TEAB stoichiometry on the monohydrolysis of **1a** using 1.2 equivalents of NaOH.

Entry	TEAB Equiv.	Time (h)	Ratio ^a^ 2a:3a	Yield ^b^(%) 2a
1	0.5	5.5	60:40	43
2	1.0	4.0	81:19	80
3	1.5	3.5	72:28	67
4	2.0	3.5	26:74	25
5	2.5	3.5	10:90	9

^a^ Ratios based on HPLC analysis; ^b^ Isolated yields of **2a** recrystallization from acetone.

In order to analyze the scope of the methodology, different substituted dicarboxylate products **1b–k** were used under the following conditions: 10% water-ethanol media, 1.2 equivalents of NaOH and 1.0 equivalent of TEAB at 40 °C, as shown in Table 6.

**Table 6:** Monohydrolysis yields for a variety of 4-aryl-4*H*-pyrans **2** using 1.2 equivalents of NaOH.

**Substrate**	R	Ratio ^a^ 2:3	Yield ^b^(%)	Melting point (°C)
**1b**	*p*-CH_3_	72:28	69	123.6–124.7
**1c**	*p*-OCH_3_	52:48	51	110.3–112.0
**1d**	*p*-Cl	69:31	66	141.8–143.1
**1e**	*m*-Cl	58:42	54	132.1–133.4
**1f**	*p*-F	75:25	69	137.4–139.1
**1g**	*p*-NO_2_	50:50	49	110.1–111.7
**1h**	*m*-NO_2_	46:54	40	121.4–121.9
**1i**	*m-*Br	65:35	62	161.4–162.7
**1j**	*m-*NHCOCH_3_	20:80	20	181.2–183.2
**1k**	2,3,4*-*triOCH_3_	45:55	42	149.1–150.4

^a^ Ratios based on HPLC analysis; ^b^ Isolated yields of **2**.

In general, the selective monohydrolysis of symmetric diesters **1** was successful and the corresponding monoesters **2** were obtained in 20–80% yields. The effects of substituents on the aryl ring of 4-aryl-4*H*-pyrans showed no discernable pattern. That is, for electron-withdrawing groups such as *p*-F and *p*-NO_2_, the yields of monoesters **2f** and **2g** (69% and 49%, respectively) with ratios of **2** and **3** (75:25 and 50:50, respectively) were similar to those of **2b** and **2c** (69% and 51%, respectively) with ratios of **2** and **3** (72:28 and 52:48, respectively), which have electron-donating groups (*p*-CH_3_ and *p*-OCH_3_, respectively). An increase in the yields of **2** was observed with the electron-withdrawing groups of *p*-Cl and *p*-NO_2_ (66% and 49%, respectively) with ratios of **2** and **3** (69:31 and 50:50, respectively) in comparison to *m*-Cl, *m*-NO_2_ (54% and 40%, respectively) with ratios of **2** and **3** (58:42 and 46:54, respectively). The lowest yield was observed with *m*-NHCOCH_3_ (**2j**) with the lowest ratio of **2j** and **3j** (20:80), likely due to its decreased solubility and susceptibility to hydrolysis of the acetyl amino group in alkaline water-ethanol media.

## 3. Experimental

### 3.1. General

Melting points were determined using an X-5 apparatus (open capillaries, uncorrected values). ^1^H- and^13^C-NMR spectra were acquired at ambient temperature (25 °C) in CDCl_3_ on a Bruker AV 400 spectrometer (400 and 100.6 MHz, respectively). Infrared spectra were recorded on a Bruker VERTEX70 instrument as potassium bromide pellets. Mass spectra were recorded on an Agilent G3250AA LC/MSD TOF system. The reaction process and the products were routinely monitored by HPLC (Waters 600, UV 2487 dual absorbance detector) with a C-18 column (4.6 × 250 mm; eluent: acetonitrile-H_2_O = 65:35; 25 °C; flow rate = 1 mL/min; UV detection at 220 nm). The chemical agents used in this study were all commercially available; diesters **1** were prepared according to our previously described method [9].

### 3.2. General Procedure for the Synthesis of Compounds 2

A mixture of **1** (1.0 mmol) and TEAB (0.36 g, 1.0 mmol) was dissolved in ethanol (20 mL) and water (1.75 mL). The reaction mixture was stirred with the evolution of heat; to this mixture was added dropwise with stirring 1.0 M NaOH aqueous solution in the amount indicated in Table 1, Table 2, Table 3, Table 4, Table 5 and Table 6 (0.1, 0.3, 0.5, 1.0, 1.2, 1.5 and 2.0 equivalents) until the mixture temperature reached 40 °C. The reaction mixture was stirred until complete consumption of the starting diester **1** was observed by TLC. The solution was then poured into water (150 mL) and acidified to pH 1–2 with 6.0 M HCl at 5–10 °C. A fine white precipitate was formed and filtered off, washed with ice water until reaching pH 7 and dried under vacuum.

*5-(ethoxycarbonyl)-2,6-dimethyl-4-aryl-4H-pyran-3-carboxylic acid* (**2a**). Yield (0.24 g, 80%), Mp 98.3–100.0 °C. IR: 705, 1191, 1626, 1664, 1709, 2372, 2981, 3432 cm^−1^. ^1^H-NMR: δ = 1.21–1.23 (t, 3 H, CH_2_CH_3_), 2.38 (s, 6 H, CH_3_), 4.10–4.13 (m, 2 H, CH_2_CH_3_), 4.75 (s, 1 H, Ar–CH), 7.15 (d, *J* = 8.8 Hz, 2 H, Ar–H), 7.27 (d, *J* = 8.8 Hz, 2 H, Ar–H), 10.40–13.05 (s, 1 H, COOH).^13^C-NMR: δ = 14.2, 15.6, 41.8, 61.7, 106.6, 107.3, 125.8, 128.7, 129.1, 142.2, 156.6, 159.4. HRMS: *m/z* [M + K]^+^ calcd for C_17_H_18_O_5_K: 341.1218; found: 341.1220.

*5-(ethoxycarbonyl)-2,6-dimethyl-4-p-tolyl-4H-pyran-3-carboxylic acid* (**2b**). Yield (0.22 g, 69%), Mp 123.6–124.7 °C. IR: 933, 1191, 1621, 1699, 2373, 2982, 3430 cm^−1^. ^1^H-NMR: δ = 1.19–1.23 (t, 3 H, CH_2_CH_3_), 2.32 (s, 3 H, CH_3_), 2.38 (s, 6 H, CH_3_), 4.09–4.11 (m, 2 H, CH_2_CH_3_), 4.75 (s, 1 H, Ar–CH), 7.10 (d, *J* = 8.8 Hz, 2 H, Ar–H), 7.15 (d, *J* = 8.8 Hz, 2 H, Ar–H), 11.00–12.98 (s, 1 H, COOH).^13^C-NMR: δ = 14.2, 15.6, 24.3, 41.8, 61.7, 106.6, 107.3, 129.0, 135.4, 139.2, 156.6, 159.4, 167.2, 171.3. HRMS: *m/z* [M + Na]^+^calcd for C_18_H_20_O_5_Na: 339.1326; found: 339.1322. 

*5-(ethoxycarbonyl)-4-(4-methoxyphenyl)-2,6-dimethyl-4H-pyran-3-carboxylic acid* (**2c**): Yield (0.17 g, 51%), Mp 110.3–112.0 °C. IR: 932, 1180, 1625, 1715, 2371, 2982, 3433 cm^−1^; ^1^H-NMR: δ = 1.12–1.24 (t, 3 H, CH_2_CH_3_), 2.36 (s, 6 H, CH_3_), 3.77 (s, 3 H, OCH_3_), 4.07–4.13 (m, 2 H, CH_2_CH_3_), 4.77 (s, 1 H, Ar–CH), 6.98 (d, *J* = 8.8 Hz, 2 H, Ar–H), 7.35 (d, *J* = 8.8 Hz, 2 H, Ar–H), 11.10–13.90 (s, 1 H, COOH).^13^C-NMR: δ = 14.1, 18.5, 19.1, 37.2, 55.1, 60.3, 107.6, 108.8, 113.4, 129.3, 137.4, 157.8, 158.3, 160.4, 166.5, 171.3. HRMS: *m/z* [M − H]^+^ calcd for C_18_H_20_O_6_: 331.1323; found: 331.1319.

*4-(4-chlorophenyl)-5-(ethoxycarbonyl)-2,6-dimethyl-4H-pyran-3-carboxylic acid* (**2d**): Yield (0.23 g, 66%), Mp 141.8–143.1 °C. IR: 853, 1180, 1655, 1718, 2372, 2983, 3436 cm^−1^; ^1^H-NMR: δ = 1.21–1.24 (t, 3 H, CH_2_CH_3_), 2.37 (s, 6 H, CH_3_), 4.09–4.13 (m, 2 H, CH_2_CH_3_), 4.71 (s, 1 H, Ar–CH), 6.87 (d, *J* = 8.8 Hz, 2 H, Ar–H), 7.27 (d, *J* = 8.8 Hz, 2 H, Ar–H), 11.3 (s, 1 H, COOH).^13^C-NMR: δ = 14.1, 18.5, 19.1, 37.2, 55.1, 60.3, 107.6, 108.8, 113.4, 129.3, 137.4, 157.8, 158.3, 160.4, 166.5, 172.2. HRMS: *m/z* [2M - CH3]^+^ calcd for C_17_H_17_ClO_5_: 687.2383; found: 687.2388.

*4-(3-chlorophenyl)-5-(ethoxycarbonyl)-2,6-dimethyl-4H-pyran-3-carboxylic acid* (**2e**): Yield (0.18 g, 54%), Mp 132.1–133.4 °C. IR: 853, 1188, 1656, 1718, 2372, 2983, 3433 cm^−1^; ^1^H-NMR: δ = 1.23–1.27 (t, 3 H, CH_2_CH_3_), 2.39 (s, 6 H, CH_3_), 4.10–4.17 (m, 2 H, CH_2_CH_3_), 4.76 (s, 1 H, Ar–CH), 7.09 (d, *J* = 8.8 Hz, 2 H, Ar–H), 7.38 (d, *J* = 8.8 Hz, 2 H, Ar–H), 10.40–13.40 (s, 1 H, COOH).^13^C-NMR: δ = 14.1, 18.5, 19.1, 37.2, 55.1, 60.3, 107.6, 108.8, 113.4, 129.3, 137.4, 157.8, 158.3, 163.4, 165.4, 176.3. HRMS: *m/z* [M − H]^+^ calcd for C_17_H_17_ClO_5_: 335.0821; found: 335.0821.

*5-(ethoxycarbonyl)-4-(4-fluorophenyl)-2,6-dimethyl-4H-pyran-3-carboxylic acid* (**2f**): Yield (0.22 g, 69%), Mp 137.4–139.1 °C. IR: 849, 1128, 1192, 1710, 2372, 2983, 3152, 3425 cm^−1^; ^1^H-NMR: δ = 1.18–1.21 (t, 3 H, CH_2_CH_3_), 2.39 (s, 6 H, CH_3_), 4.06–4.11 (m, 2 H, CH_2_CH_3_), 4.72 (s, 1 H, Ar–CH), 6.98 (d, *J* = 8.8 Hz, 2 H, Ar–H), 7.25 (d, *J* = 8.8 Hz, 2 H, Ar–H), 11.70–12.80 (s, 1 H, COOH).^13^C-NMR: δ = 14.1, 18.5, 19.2, 37.3, 60.4, 107.4, 108.5, 114.7, 114.9, 129.8, 129.9, 140.8, 158.3, 160.4, 162.8, 166.3, 172.3. HRMS: *m/z* [M – CH_3_]^+^ calcd for C_17_H_17_FO_5_: 305.1561; found: 305.1556.

*5-(ethoxycarbonyl)-2,6-dimethyl-4-(4-nitrophenyl)-4H-pyran-3-carboxylic acid* (**2g**): Yield (0.16 g, 49%), Mp 110.1–111.7 °C. IR: 874, 1167, 1347, 1624, 1721, 2349, 2938, 3077, 3462 cm^−1^. ^1^H-NMR: δ = 1.18–1.21 (t, 3 H, CH_2_CH_3_), 2.39 (s, 6 H, CH_3_), 4.06–4.11 (m, 2 H, CH_2_CH_3_), 4.84 (s, 1 H, Ar–CH), 7.26 (d, *J* = 8.8 Hz, 2 H, Ar–H), 7.42 (d, *J* = 8.8 Hz, 2 H, Ar–H), 10.90–13.20 (s, 1 H, COOH).^13^C-NMR: δ = 14.1, 18.6, 19.2, 38.3, 60.7, 106.4, 107.5, 123.4, 129.3, 146.7, 152.3, 159, 161.7, 165.8, 171.5. HRMS: *m/z* [M − H]^+^ calcd for C_17_H_17_NO_7_: 335.0831; found: 335.0830. 

*5-(ethoxycarbonyl)-2,6-dimethyl-4-(3-nitrophenyl)-4H-pyran-3-carboxylic acid* (**2h**): Yield (0.13 g, 40%), Mp 121.4–121.9 °C. IR: 900, 1221, 1348, 1652, 1703, 2372, 2952, 3091, 3348 cm^−1^. ^1^H-NMR: δ = 1.19–1.22 (t, 3 H, CH_2_CH_3_), 2.30 (s, 6 H, CH_3_), 4.11–4.17 (m, 2 H, CH_2_CH_3_), 4.89 (s, 1 H, Ar–CH), 7.21 (d, *J* = 8.8 Hz, 2 H, Ar–H), 7.42 (d, *J* = 8.8 Hz, 2 H, Ar–H), 10.84-12.40 (s, 1 H, COOH).^13^C-NMR: δ = 14.1, 18.6, 19.2, 38.6, 62.7, 106.4, 107.5, 123.4, 129.3, 146.7, 152.3, 160.3, 162.7, 165.8, 175.4. HRMS: *m/z* [M]^+^ calcd for C_17_H_17_NO_7_: 336.0815; found: 336.0816.

*4-(3-bromophenyl)-5-(ethoxycarbonyl)-2,6-dimethyl-4H-pyran-3-carboxylic acid* (**2i**): Yield (0.24 g, 62%), Mp 161.4–162.7 °C. IR: 900, 1221, 1380, 1628, 1700, 2372, 2952, 3091, 3348 cm^−1^. ^1^H-NMR: δ = 1.22–1.25 (t, 3 H, CH_2_CH_3_), 2.34 (s, 6 H, CH_3_), 4.09–4.15 (m, 2 H, CH_2_CH_3_), 4.73 (s, 1 H, Ar–CH), 7.10 (s, *J* = 8.8 Hz, 2 H, Ar–H), 7.15–7.35 (m, *J* = 8.8 Hz, 3 H, Ar–H), 11.40–13.20 (s, 1 H, COOH).^13^C-NMR: δ = 14.2, 17.6, 28.3, 38.2, 61.7, 106.6, 107.3, 125.4, 128.3, 146.2, 156.3, 159.4, 160.7, 165.4, 174.3. HRMS: *m/z* [M + Na]^+^ calcd for C_17_H_17_BrO_5_Na: 403.0315; found: 403.0315.

*4-(3-acetamidophenyl)-5-(ethoxycarbonyl)-2,6-dimethyl-4H-pyran-3-carboxylic acid* (**2j**): Yield (0.07 g, 20%), Mp 181.2–183.2 °C. IR: 888, 1188, 1330, 1652, 1717, 2345, 2983, 3061, 3425 cm^−1^. ^1^H-NMR: δ = 1.20–1.24 (t, 3 H, CH_2_CH_3_), 2.16 (s, 3 H, NHCOCH_3_), 2.34 (s, 6 H, CH_3_), 4.07–4.14 (m, 2 H, CH_2_CH_3_), 4.92 (s, 1 H, Ar–CH), 6.94–7.23 (m, 4 H, Ar–H), 7.44 (s, 1 H, NHCOCH_3_), 11.60–13.50 (s, 1 H, COOH).^13^C-NMR: δ = 14.2, 18.6, 22.9, 41.8, 60.7, 106.6, 107.3, 118.4, 124.3, 128.7, 138.3, 142.4, 156.6, 165.8, 167.2, 173.3. HRMS: *m/z* [M + Na]^+^ calcd for C_19_H_21_NO_6_Na: 382.1464; found: 382.1455.

*5-(ethoxycarbonyl)-2,6-dimethyl-4-(2,3,4-trimethoxyphenyl)-4H-pyran-3-carboxylic acid* (**2k**): Yield (0.16 g, 42%), Mp 149.1–150.4 °C. IR: 793, 1048, 1147, 1189, 1291, 1397, 1494, 1645, 1724, 2935, 3037, 3444 cm^−1^. ^1^H-NMR: δ = 1.21–1.25 (t, 3 H, CH_2_CH_3_), 2.32 (s, 6 H, CH_3_), 3.84 (t, 9 H, OCH_3_), 4.08–4.16 (m, 2 H, CH_2_CH_3_), 4.77 (s, 1 H, Ar–CH), 7.20 (d, *J* = 8.8 Hz, 1 H, Ar–H), 7.34 (d, *J* = 8.8 Hz, 1 H, Ar–H), 10.95-12.70 (s, 1 H, COOH).^13^C-NMR: δ = 14.2, 17.6, 41.7, 56.5, 61.7, 106.6, 107.5, 113.4, 128.4, 139.1, 145.8, 157.6, 167.2, 171.3, 174.4. HRMS: *m/z* [M + Na]^+^calcd for C_21_H_27_O_8_Na: 415.1527; found: 415.1526.

## 4. Conclusions

In summary, experiments were designed to study and optimize the selective monohydrolysis of symmetric diethyl 2,6-dimethyl-4-aryl-4*H*-pyran-3,5-dicarboxylate diesters **1** for the preparation of 5-(ethoxycarbonyl)-2,6-dimethyl-4-aryl-4*H*-pyran-3-carboxylic acid monoesters **2**. The main parameters that affected the yields of monoesters **2** were investigated, and the optimal conditions were established as a 10% water-ethanol media in the presence of a 1.0 equiv. TEAB, with the following molar ratios for NaOH: **1a** = 1.2:1 at 40 °C. This reaction represents a unique, flexible and simple method for the monohydrolysis of diesters to afford monoesters in 20–80% yields under the optimized condition.
